# A Multivariate Approach to Ethnopharmacology: Antidiabetic Plants of Eeyou Istchee

**DOI:** 10.3389/fphar.2021.511078

**Published:** 2022-01-18

**Authors:** Braydon Hall, Michel Rapinski, Danielle Spoor, Hoda Eid, Ammar Saleem, John T. Arnason, Brian Foster, Alain Cuerrier, Pierre S. Haddad, Cory S. Harris

**Affiliations:** ^1^ Canadian Institutes of Health Research (CIHR) Team in Aboriginal Anti-Diabetic Medicines, Montréal, QC, Canada; ^2^ Department of Biology, University of Ottawa, Ottawa, ON, Canada; ^3^ Department of Pharmacology and Physiology, CIHR Team in Aboriginal Anti-Diabetic Medicines, Université de Montréal, Montréal, QC, Canada; ^4^ Department of Pharmacognosy, Beni-Suef University, Cairo, Egypt

**Keywords:** diabetes, medicinal plants, indigenous knowledge, James Bay Cree, *in vitro* bioassays

## Abstract

An ethnopharmacological metanalysis was conducted with a large database available on antidiabetic activities of plant foods and medicines from the northern boreal forest, which are traditionally used by the indigenous Cree of James Bay, Quebec, Canada. The objective was to determine which bioassays are closely associated with the traditional knowledge of the Cree and which pharmacological metrics and phytochemical signals best define these plants and their groups. Data from 17 plant species, ethnobotanically ranked by syndromic importance value for treatment of 15 diabetic symptoms, was used along with 49 bioassay endpoints reported across numerous pharmacological studies and a metabolomics dataset. Standardized activities were separated into primary, secondary and safety categories and summed to produce a Pharmacological Importance Value (PIV) in each of the three categories for each species. To address the question of which pharmacological metrics and phytochemical signals best define the CEI anti-diabetes plants, multivariate analyses were undertaken to determine groupings of plant families and plant parts. The analysis identified *Larix larcina* as the highest PIV species in primary assays, *Salix planifolia* in secondary assays, and *Kalmia angustifolia* in safety assays, as well as a ranking of other less active species by PIV. Multivariate analysis showed that activity in safety PIV monitored mainly with cytochrome P450 inhibition patterns best reflected patterns of traditional medicine importance in Cree traditional knowledge, whereas potent primary bioactivities were seen in individual plants determined to be most important to the Cree for anti-diabetes purposes. In the secondary anti-diabetes assays, pharmacological variability was better described by plant biology, mostly in terms of the plant part used. Key signal in the metabolomics loadings plots for activity were phenolics especially quercetin derivatives. Traditional Indigenous knowledge in this analysis was shown to be able to guide the identification of plant pharmacological qualities in scientific terms.

## Introduction

Due to a better understanding of the consequences of loss of indigenous culture through world development, a recent focus of the scientific community is the medicinal value of traditional knowledge (Schultes, 1994; Fabricant and Farnsworth, 2001). Previously, research on indigenous plant use often gave little depth of focus to the medicinal uses (Core, 1967; Turner and Bell, 1971) or focused only on specific plants (Rymer, 1976; Chandler et al., 1982). As the field of ethnobotany grew, studies began shifting from purely descriptive and qualitative approaches toward quantitative methods aiming to sift through the vast yet newly documented plant knowledge of indigenous peoples (Prance, 1991; Höft et al., 1999). The primary intention of these statistical approaches was to determine similarity of patterns between plants or the people that use them. These methods have been explored in the context of consensus and significance of use but have never been extended to include plant bioactivity and pharmacology.

Developing a unique combination of community and laboratory methods in the analysis of traditional knowledge data, the Team in Aboriginal Anti-diabetic Medicine (TAAM) was assembled in 2003 by Dr. Pierre Haddad of the University of Montreal and funded by the Canadian Institutes of Health Research (CIHR) to address the issue of rising rates of Type 2 diabetes (T2D) in Canadian First Nations communities. The work of the TAAM focused mostly on the Cree of Eeyou Istchee (CEI), a group of communities located in the James Bay region of Northern Québec. The team included researchers from three major Canadian research universities and community stakeholders representing both Elders and Public Health professionals. The goal was to answer a call from Cree Elders to their leadership to revitalize traditional medicine as an accessible and culturally relevant method of T2D prevention and management. Through multi-institutional collaborative work, the TAAM investigated the ethnobotany, pharmacology, and phytochemistry of traditional Northern Cree medicinal plants in the context of T2D.

After more than 15 years of data collection and analysis focusing on 17 medicinal plant species prioritized based ethnobotanical use for treating symptoms of diabetes (Table 1), the TAAM was in a unique position to ask important yet previously unexplored ethnopharmacological questions. First, which bioassays are closely associated with the traditional knowledge of the CEI? Second, what pharmacological metrics and phytochemical signals best define these plants and their groups? To investigate these questions, we applied multivariate analyses across an interdisciplinary yet cohesive data set and explored how the anti-diabetic bioassay results relate ethnobotanical knowledge, chemistry, and biology of CEI medicinal plants.

**TABLE 1:** The 17 most important traditional medicinal plants of more than 50 plants mentioned for the treatment of diabetic symptoms and complications among the Cree of Eeyou Istchee as identified through interviews with elders.

Plant	Cree name	Family	Part	SIV	Rank^b^
*Abies balsamea* (L) Mill	Inaasht	Pinaceae	Bark	0.0244	8
*Alnus incana subsp. rugose* (Du Roi) R. T	Atushpi	Betulaceae	Bark	0.0239	9
*Gaultheria hispidula* (L) Muhl	Pieuminaan	Ericaceae	Leaves	0.0100	16
*Juniperus communis* L	Kaahkaachiiminaahtikw	Cupressaceae	Berries	0.0203	10
*Kalmia angustifolia* L	Uishichipukw	Ericaceae	Leaves	0.0321	6
*Larix laricina* Du Roi (K. Koch)	Watnagan	Pinaceae	Bark	0.0369	2
*Lycopodium clavatum* L	Pashtnahoagin	Lycopodaceae	Whole	0.00866	17
*Picea glaucaa* (Moench.) Voss	Minhikw	Pinaceae	Leaves	0.0347	5
*Picea mariana* (P. Mill) BSP	Inaahtikw	Pinaceae	Cones	0.0352	4
*Pinus banksiana* Lamb	Ushchishk	Pinaceae	Cones	0.0179	11
*Populus balsamifera* L	Miitus	Salicaceae	Bark	0.0117	15
*Rhododendron groenlandicum* (Oeder) Kron and Judd	Kachichepukw	Ericaceae	Leaves	0.0377	1
*Rhododendron tomentsum* (Harjama)	Wiisichipukw	Ericaceae	Leaves	0.0359	3
*Salix planifolia* Pursh	Pieuatikw	Salicaceae	Bark	0.0178	12
*Sarracenia purpurea* L	Ayigadash	Sarracenaceae	Whole^a^	0.0132	14
*Sorbus decora* (Sarg.) C. K. Schneid	Mushkuminanatikw	Rosaceae	Bark	0.0312	7
*Vaccinium vitis-idaea* L	Wishichimna	Ericaceae	Berries	0.0164	13

Syndromic Importance Value (SIV) indicates the degree of association with uses related to treating diabetes symptoms where larger values indicate stronger association (Leduc et al., 2006).

a Above ground material only.

b Ranked with 1 = most important.

## Methods

### Data Collection and Assembly

Since 2003, the TAAM has collected data on the use of medicinal plants from the CEI for the prevention and management of T2D. This research began with a targeted ethnopharmacological approach and novel statistical methods to evaluate the CEI ethnobotany with specific focus on traditional medicines used for treating major symptoms of T2D (Leduc et al., 2006; Harbilas et al., 2009). All reported medicines were ranked according to Syndromic Importance Value (SIV), which considers both the frequency of reported use by elders for specific diabetic symptoms and the clinical importance of these symptoms as determined by a panel of diabetes specialists (Leduc et al., 2006). Once a subset of 17 plant medicines was prioritized to assess their anti-diabetic potential (Table 1), chemical and bioassay data was assembled evaluating the plant extracts for phytochemical composition and biological activities in dozens of bioassays, respectively. The latter were subdivided into bioassays evaluating primary antidiabetic potential (those related to glucose and lipid homeostasis), secondary antidiabetic potential (those related to complications arising from glucolipotoxicity), and safety (those related to potential herb-drug interactions). It is this cumulative set of ethnobotanical, phytochemical and pharmacological data that served as the basis for the statistical analyses presented here.

### Plant Extracts

The 17 medicinal Cree plants were collected under the guidance of Cree elders over the course of more than a decade of collaboration, in the CEI territory of Northern Québec, Canada (Spoor et al., 2006; Harbilas et al., 2009; Haddad et al., 2012). Briefly, dried plant material was ground and extracted in 80% ethanol (10 ml/g dry material) twice for 24 h on a mechanical shaker. Extracts were then filtered, combined, lyophilized, and stored at 4°C, before being reconstituted in vehicle solvent as needed for study. Standard operating procedures were put in place to ensure that all participating laboratories used identical study material and reconstituted dry extracts in a uniform way before testing in bioassays.

### Pharmacology Dataset

In total, 69 bioassay endpoints reported across numerous pharmacological studies were included in this investigation. Of those, 49 contained complete data for all 17 plant species. A full breakdown of the individual endpoints can be found in Supplementary Material. To deal with the great diversity of experimental methods and variable distribution of data relative to control and plant treatments in each bioassay, data were standardized to the most active plant relative to the negative solvent control (generally 0.1% DMSO); as such, the most active plant had a standardized value of 1 and less active plants had standardized values between 1 and the negative vehicle control, set to 0. In cases where plants exhibited activity considered opposite to an anti-diabetes activity, for example inhibition of hepatic glucose uptake instead of stimulation, any plant data exhibiting this activity were collapsed to the value of the negative solvent control, namely, zero.

An effort was also made to estimate a given plant’s overall pharmacological activity in each set of bioassays representing primary antidiabetic potential, secondary antidiabetic potential or herb-drug interaction potential, as described above. For that purpose, the standardized activity described in the previous paragraph was summed over each set of bioassays and then divided by the number of bioassays in this set. In this way, a plant that would be the absolute best in all bioassays of a given set would obtain a value of 1, one that would display half of that maximal activity in the same bioassays would get a value of 0.5 and so on. We termed this value Pharmacological Importance Value (PIV) as a parallel to the SIV obtained from traditional knowledge.

### Phytochemistry Dataset

In addition to the pharmacological data, phytochemical data collected for Shang et al. (2015) on the 17 Cree medicinal plant extracts were re-analysed and included in the assembled data set and related multivariate analyses. Untargeted metabolomics analysis of plant extracts (1 mg/ml in DMSO) was performed using Ultra-High Pressure Liquid Chromatography with Quantitative Time-of-Flight (UPLC/QTOF-MS) equipped with electrospray ionization (ESI) interface (Xevo G2, Waters Inc.). The data were acquired and processed with MassLynx (version 4.1) and MarkerLynx (version 8.03) software. The retention times and the protonated masses were generated at a noise threshold of 500 counts and no smoothing was applied.

Raw metabolomics data is inherently three dimensional, containing values for signal mass, signal intensity and retention time, and so was condensed here to simplify analysis. Raw data also contains baseline noise signals that are not relevant to analysis and must be filtered out. Further, Waters’ proprietary data format combines normal peak data with lockmass standardization data and signal fragmentation data, which must also be filtered out. To adapt metabolomics data for this study, the fragmentation signal data was removed from the raw data file using ProteoWizard (Chambers et al., 2012). Data processing was executed primarily using MZMine (Pluskal et al., 2010), where linear normalization to the total raw peak area data was applied and thresholds were set to minimize the incorporation of signal noise. Major peaks within a retention time threshold of 0.01 min were then identified and aligned across the samples, with subsequent extraction of relevant peak data (retention time, mass-charge ratio and peak area). The exported data was evaluated to remove signals associated with DMSO blanks that were also injected. The final data matrix consisted of signals denoted by both their mass-to-charge ratio (*m/z*) and retention time as well as the intensity (peak area) of that signal for each of the 17 Cree plants.

### Data Analysis

#### Ethnopharmacological Meta-Analysis

To address the question of what biological activities associate with the traditional knowledge of the CEI, the plants were first grouped into quartiles based on their SIV values from the original CEI ethnobotany study of the TAAM (Leduc et al., 2006). The first quartile consisted of plants from ranking 1 to 4 (Table 1), therefore consisting of the four most important CEI plants relating to diabetes symptom treatment. Quartile two contained plants from rank 5–8, quartile three contained plants from rank 9–12, and quartile four contained plants from rank 13–17. These groupings were then applied to a principal component analysis (PCA) of the raw standardized pharmacological data to evaluate to what extent the traditional knowledge SIV quartiles separated. This was applied to the full pharmacological data set as well as the data sets for the individual assay categories (primary, secondary, safety).

In addition, a Pharmacological Importance Value (PIV; described in *Pharmacology Dataset* above) was generated for each plant to represent its overall level of activity in the bioassays and bioassay subcategories (Table 2). This was done by taking the average of each plants’ data from each category. In that way, plants could be compared more generally between the most and least active by way of largest and smallest PIV, respectively. These PIV values were then used in correlation analysis with SIV values to evaluate trends between the plants identified as more important by CEI elders and plants determined as being more active in bioassay analysis.

**TABLE 2:** Pharmacological importance value (PIV) of 17 CEI medicinal plants.

Species	Pharmacological importance value		
	Primary PIV	Secondary PIV	Safety PIV
*A. balsamea*	0.478	0.437	0.638
*A. incana*	0.455	0.442	0.420
*G. hispidula*	0.280	0.532	0.485
*J. communis*	0.209	0.345	0.585
*K. angustifolia*	0.163	0.503	0.723
*L. laricina*	0.495	0.587	0.627
*L. clavatum*	0.107	0.416	0.460
*P. glauca*	0.144	0.584	0.472
*P. mariana*	0.300	0.443	0.635
*P. banksiana*	0.389	0.506	0.674
*P. balsamifera*	0.115	0.439	0.251
*R. groenlandicum*	0.392	0.513	0.814
*R. tomentosum*	0.186	0.617	0.622
*S. planifolia*	0.231	0.595	0.596
*S. purpurea*	0.509	0.448	0.337
*S. decora*	0.318	0.462	0.692
*V. vitis-idaea*	0.432	0.358	0.131

Values were determined through averaging the standardized activity for plants on sets of associated bioassays.

#### Pharmacological and Phytochemical Analyses

To address the question of what pharmacological metrics and phytochemical signals best define the CEI anti-diabetes plants, multivariate analyses were then focused on groupings of plant families and plant parts. For both the pharmacology and phytochemistry datasets, PCA was completed using the plant species as the samples and the bioassays or metabolomic signal markers as variables, respectively.

To further evaluate metabolomic distributions, a hierarchal cluster analysis on the signal intensity data was performed with linkage through unweighted pair group method with arithmetic mean (UPGMA). Groupings by both plant family and plant part used were applied. To investigate key differences in the distributions of metabolic signals between discrete groups, orthogonal partial least square discriminant analysis (OPLS-DA) was used to generate S-plots where the discriminating signals between groups were taken to represent potential biomarkers. Such signals were searched using the Metlin. scripps online database to yield tentative identifications. Search parameters included the signal mass, a mass tolerance of 10 PPM, and adduct possibilities of +H, +Na, and -H2O. Tentative identifications were then based off of the most abundant compound class with lowest mass difference.

## Results

### Ethnopharmacological Meta-Analysis

**FIGURE 1:**
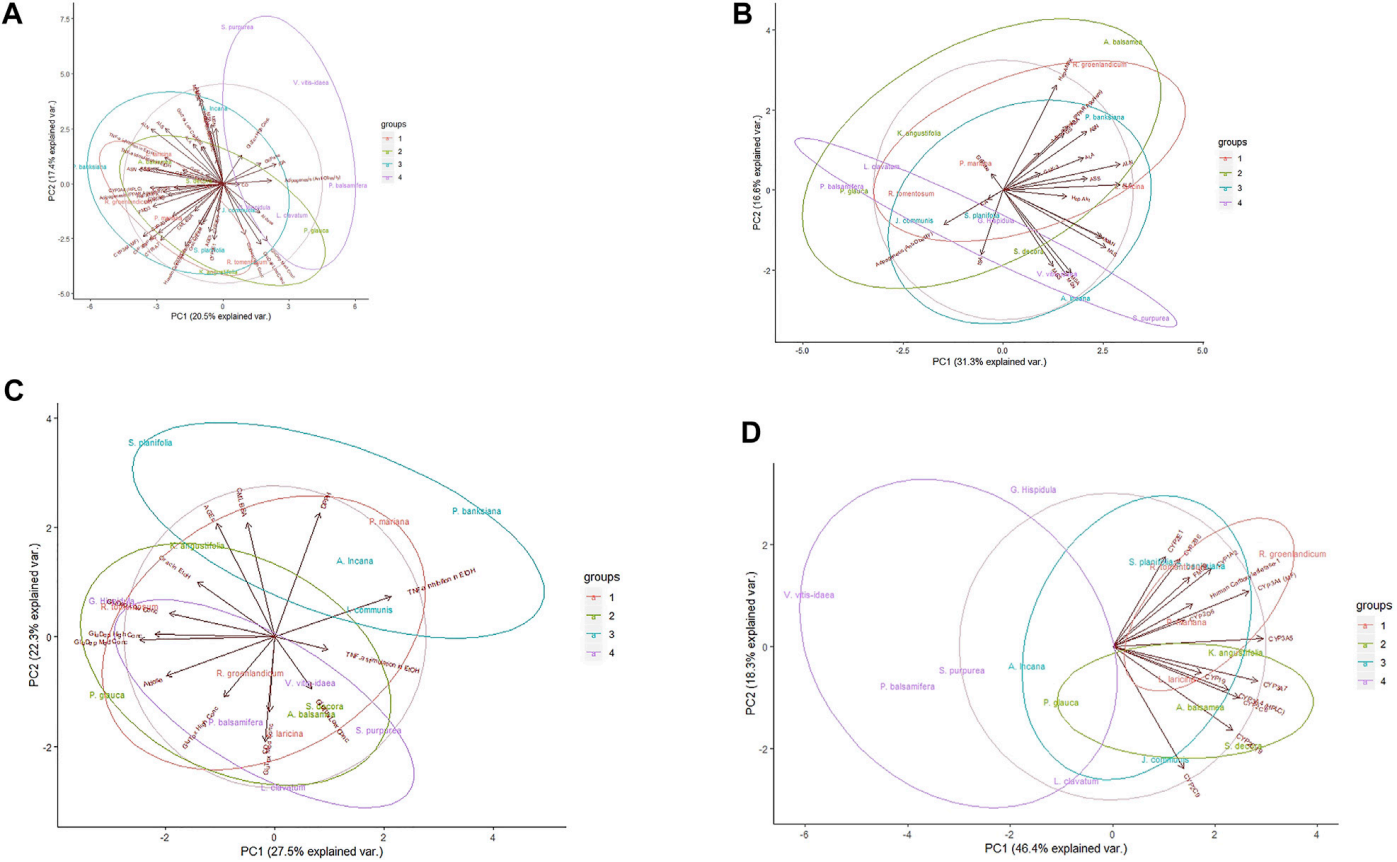
Principal component analysis of Cree medicinal plant biological activity subgroups. Groupings reflect rankings based on Syndromic Importance Values assigned to each plant through interviews with Cree elders; groups quartiles refer to the degree of relationship of plant use with diabetes symptoms. **(A)** Analysis using all evaluated bioassays. **(B)** Analysis using primary anti-diabetic bioassays. **(C)** Analysis using secondary anti-diabetic bioassays. **(D)** Analysis using herb-drug interaction bioassays. Central circles represent 95% confidence interval on full data sets. Ellipses indicate 95% confidence intervals on groupings.

Upon evaluation of all pharmacological data (Figure 1A), PCA analysis revealed a large amount of variation inherent to this data set. The first principal component (PC) only explained 20.5% of the variance, and less than 40% variance was explained by both axes. Moreover, the highly localized distribution of the cytochrome P450 (CYP) enzyme inhibition assay loading vectors (lower left quadrant) suggests that approaching all the data at the same time is sub-optimal. However, some separation of the plants, notably between the first and last SIV quartiles, respectively marked in red and purple, can be observed.

Based on overlap between the 95% confidence regions of SIV quartiles, it became clear that of the three assay types (Figures 1B–D), the safety assay data displayed the most distinctive clustering, both on its own and with respect to the groupings by traditional knowledge, especially the complete segregation of plants from quartiles 1 and 4 along PC1. Regardless of grouping, the evaluation of activity in safety assays explains the greatest amount of variation (64.7%) compared to the primary (47.9%) or secondary (49.8%) antidiabetic assays.

**FIGURE 2:**
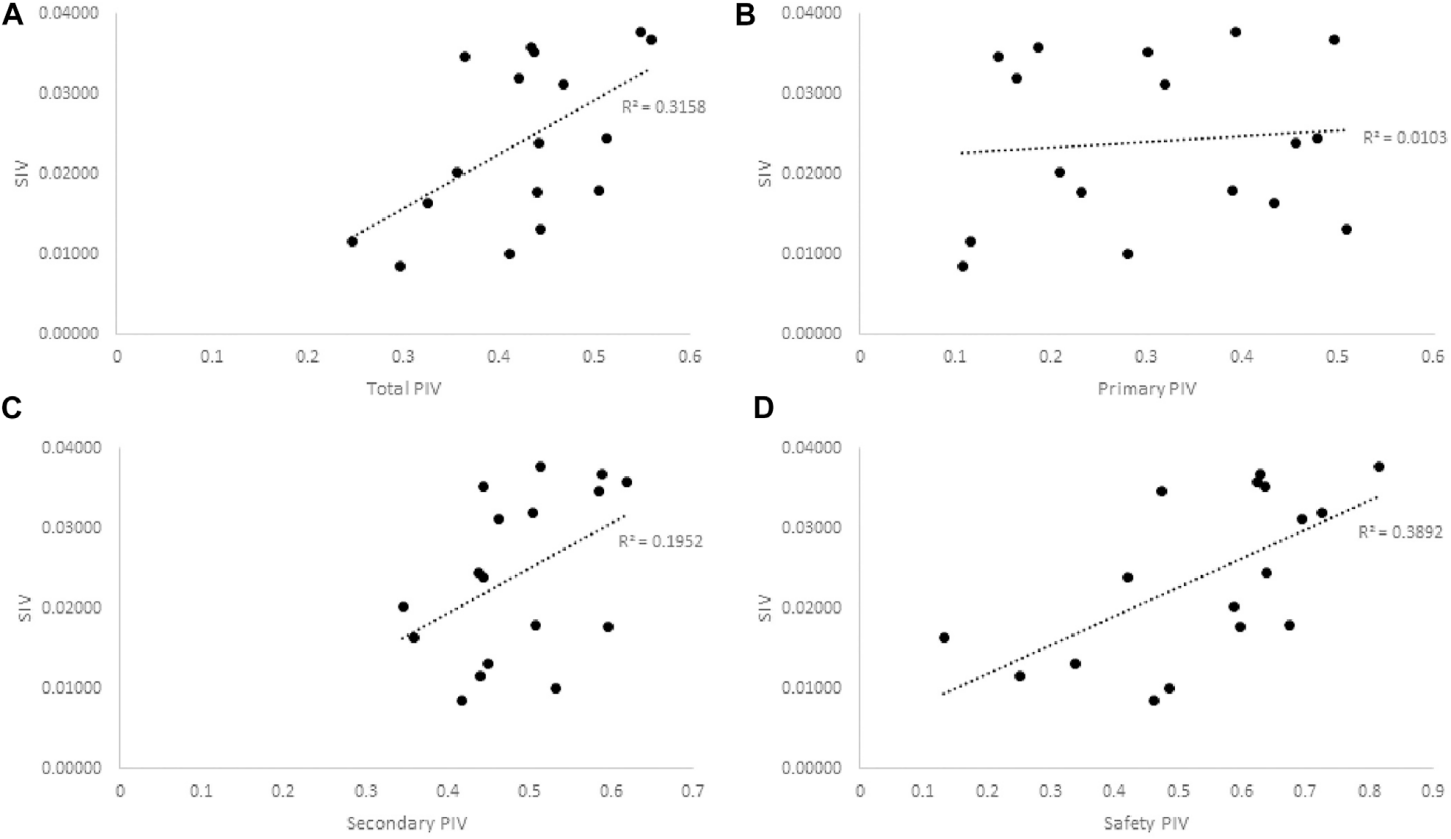
Pearson correlation plots of the SIV and PIV of Cree medicinal plants bioactivity in **(A)** All assays, *r* ^2^ = 0.3158, *p* = 0.019, **(B)** Primary anti-diabetes Assays, *r* ^2^ = 0.0103, *p* = 0.7, **(C)** Secondary anti-diabetes Assays, *r* ^2^ = 0.1952, *p* = 0.076, and **(D)** Safety assays, *r* ^2^ = 0.3892, *p* = 0.0074. Greater SIV values indicate greater associated anti-diabetic potential based on ethnobotanical evidence. Greater PIV values indicate more significant relative activity in associated bioassays.

Assessing potential correlations between the traditional knowledge (SIV) and strength of plant activity in the bioassays (PIV), similar patterns as seen with PCA emerged (Figure 2). Once again, the strongest association to the traditional knowledge and significance of use lies with the activity in CYP inhibition assays, where the plants that displayed the most and/or strongest inhibition of CYP enzymes tended to also be the ones most valued by the CEI elders.

### Pharmacological Analysis

Activity distributions among the plants were also be evaluated based on biological relationships, namely plant family and plant part. Within the primary assay data, neither plant family nor plant part relate well with the distribution of plant bioactivities (data not shown). Instead, there is a clear separation in the variable distributions for the two groups of glucose uptake stimulation (muscle and adipose tissue) from the other assays, as well as a loose grouping of the assays involving hepatocytes.

**FIGURE 3:**
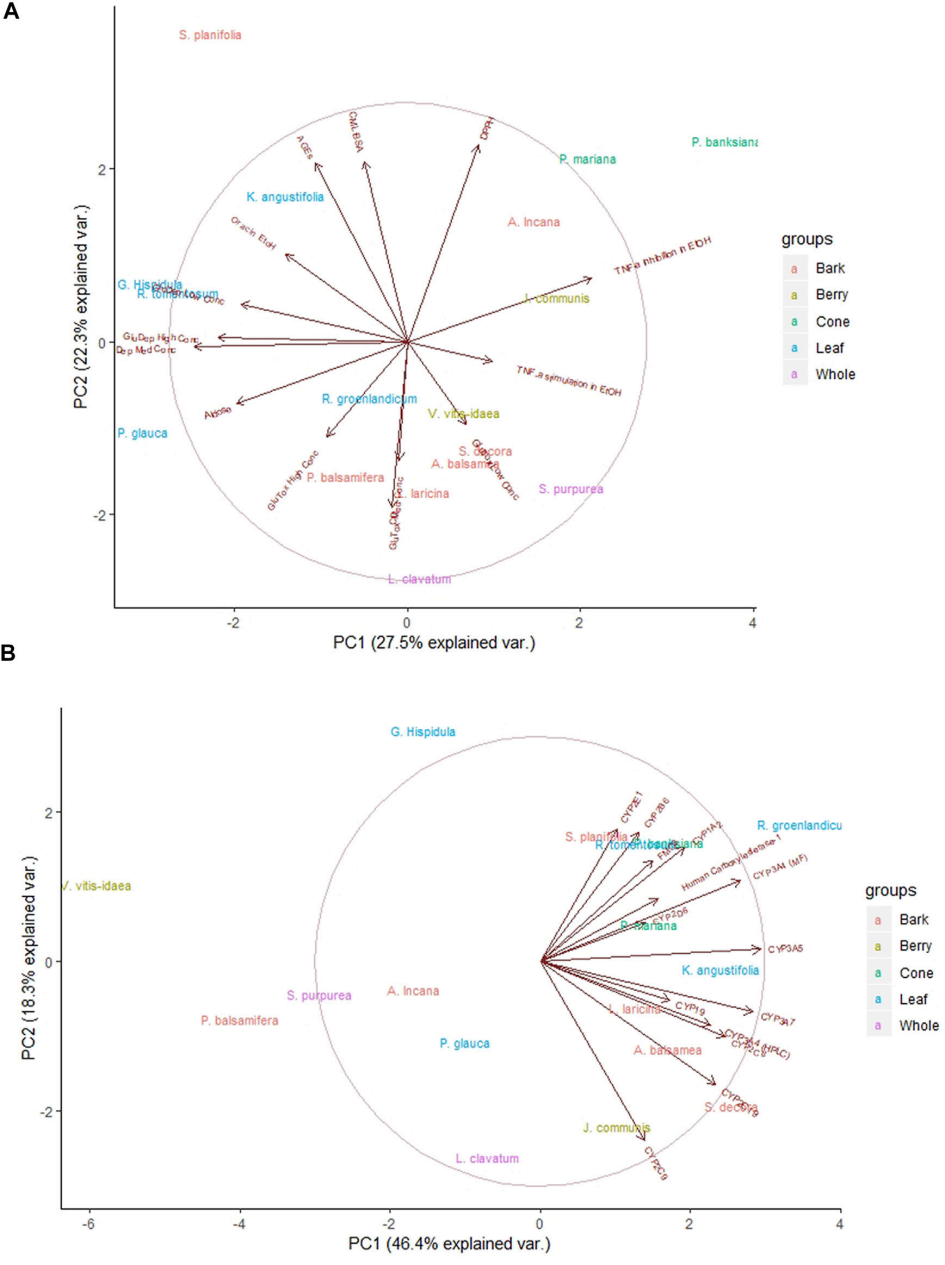
Principal component analysis of Cree medicinal plant biological activity subgroups. Groupings reflect rankings based on plant part used. **(A)** Analysis using secondary anti-diabetic bioassays. **(B)** Analysis using herb-drug interaction bioassays. Central circles represent 95% confidence interval on full data sets. Ellipses indicate 95% confidence intervals on groupings.

The main notable grouping was of leaf extracts in the secondary assay data (Figure 3A). The leaves of *R. groenlandicum*, *R. tomentosum*, *G. hispidula*, *K. angustifolia* (Ericaceae species), and *P. glauca* (needles, Pinaceae) appear to have a high degree of similarity with respect to their antioxidant and antiglycation activities, both of which relate to radical scavenging capacity. In addition, the two cone extracts, *P. banksiana* and *P. mariana*, exhibited similar bioactivity patterns in both the secondary (Figure 3A) and safety (Figure 3B) assay data.

**FIGURE 4:**
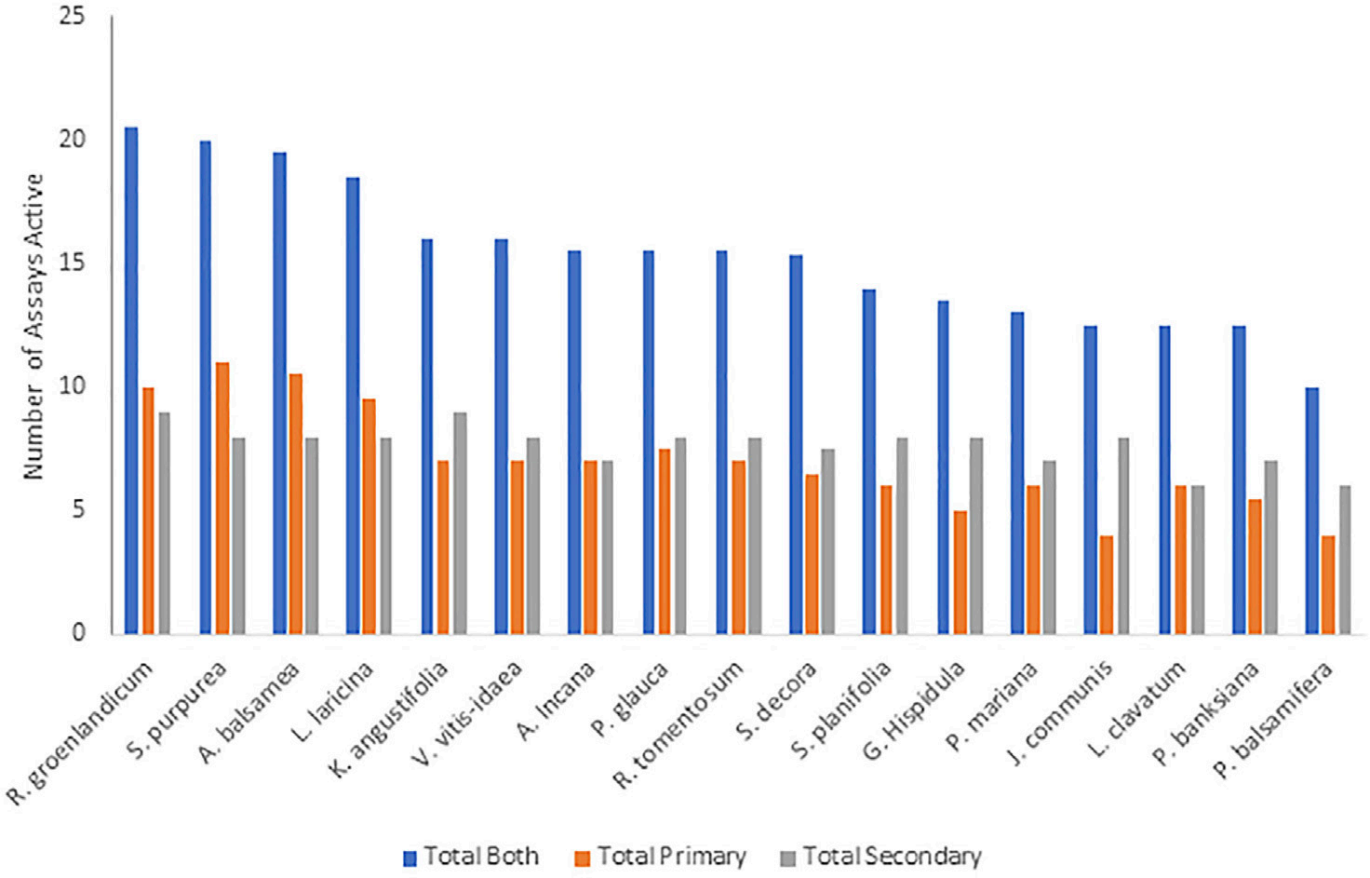
The total number of assays for which Cree medicinal plants were determined to have statistically significant activity on bioassays evaluating effects on primary and secondary anti-diabetes bioassays. All species were tested on the same set of assays.

Considering an active-inactive binary for each plant on each bioassay instead of rank also provided valuable insight. By summing the total number of assays in which statistically significant activity was observed in the previous studies (Figure 4), *R. groenlandicum, S. purpurea, A. balsamea,* and *L. laricina* initially appear to be the most biologically active species. The grouping of these 4 plants was seen through their combined activity across both sets of bioassays (primary and secondary), although this distinction appears to be more driven by the results of the primary bioassays specifically.

### Phytochemical Analysis

**FIGURE 5:**
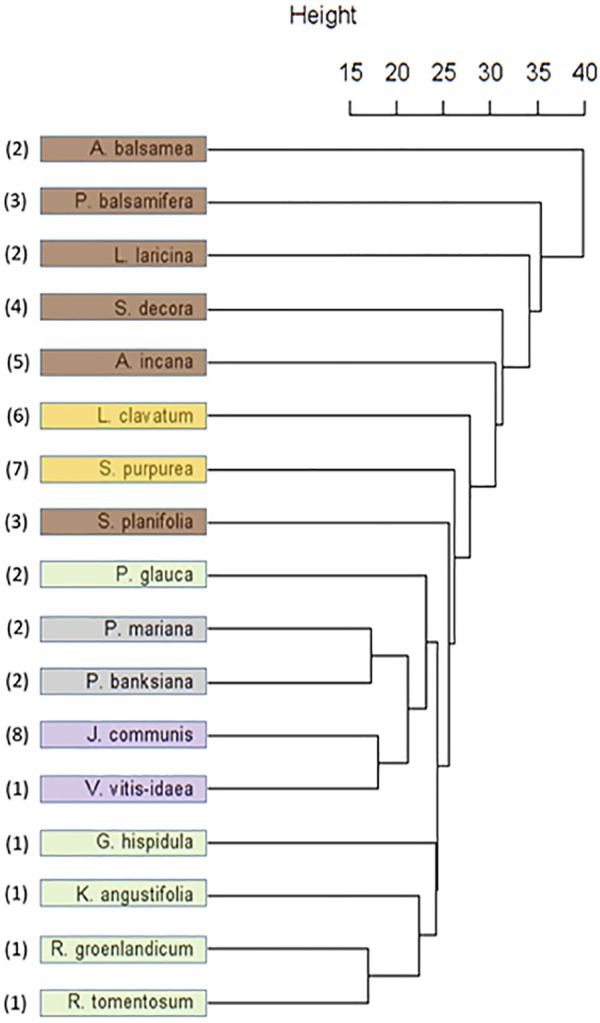
Dendrogram resulting from Unweighted Pair Group Method with Arithmetic Mean (UPGMA) cluster analysis of Cree medicinal plant metabolomes. Species colouring indicates the plant part investigated: Brown (Bark), Yellow (Whole), Green (Leaf), Grey (Cone), Purple (Berry). Numbers indicate species family: 1 (Ericaceae), 2 (Pinaceae), 3 (Salicaceae), 4 (Rosaceae), 5 (Betulaceae), 6 (Lycopodaceae), 7 (Sarracenaceae), 8 (Cupressaceae).

Hierarchal cluster analysis of the Cree metabolomics data (Figure 5) presented several distinct groupings. The groupings on the dendrogram can be seen to be more aligned with the plant parts that were tested than with the associated plant families and there was no alignment with SIV groupings (data not shown). Distinct clusters appeared with the leaves of the Ericaceae, and with the categories ofberries and cones, both considered plant fruiting bodies. There was a distinct lack of grouping among the bark samples.

**FIGURE 6:**
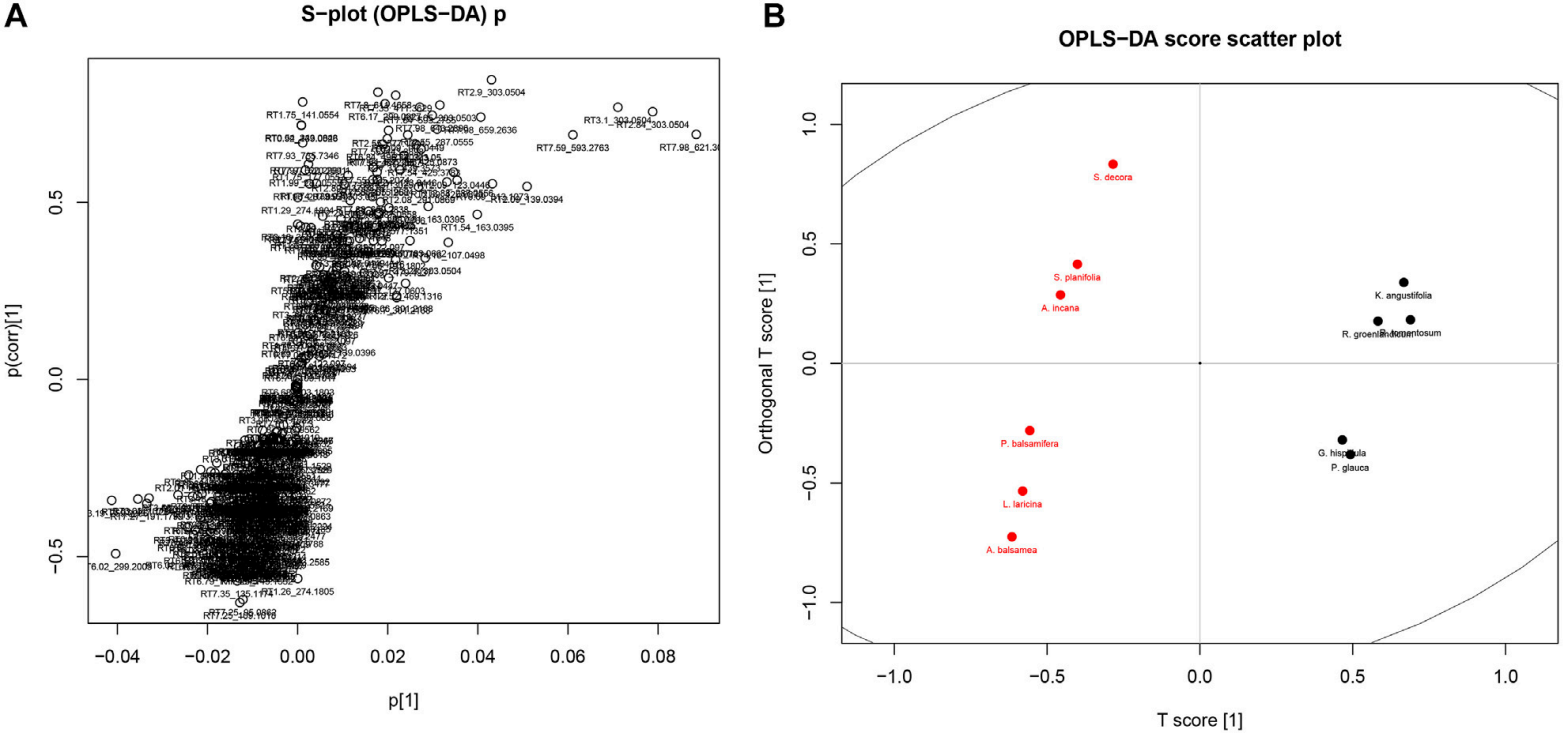
OPLS-DA of Cree medicinal plants metabolomics, grouped by specific plant parts, namely leaves (black) and barks (red). **(A)** Distribution of metabolomics signal loadings. **(B)** Distribution of plant scores.

**FIGURE 7:**
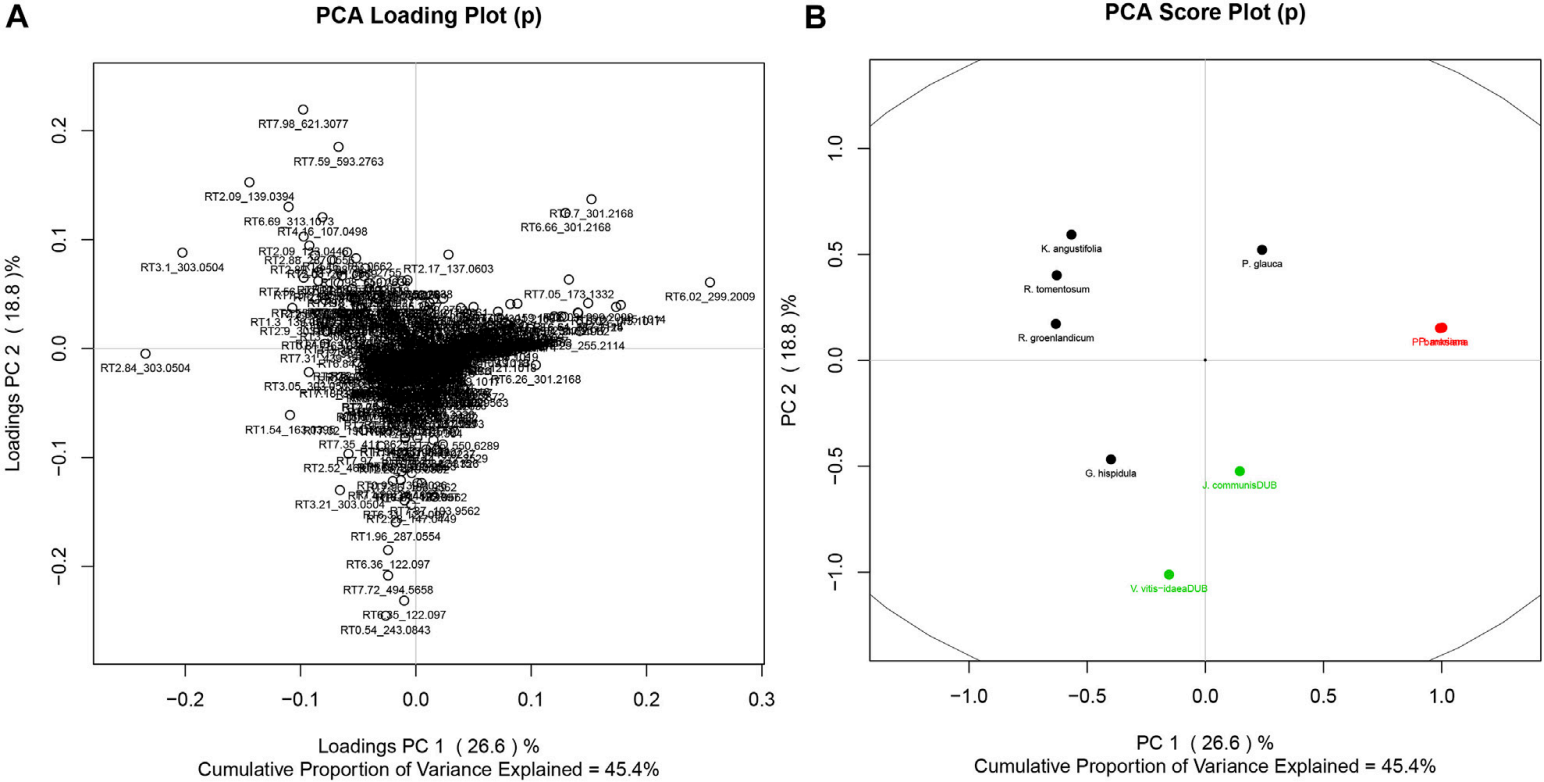
PCA of Cree medicinal plants, grouped by specific plant parts, namely leaves (black), berries (green), and cones (red). **(A)** PCs 1 and 2 for the metabolomics signal loadings. **(B)** PCs 1 and 2 for plant scores.

Given the similarity in the phytochemistry of the leaves, discriminant analysis and PCA were used to explore what chemical signals appear to be most representative of this group compared to the rest of the Cree plants. Separate analyses were completed to compare the leaves to their least similar group, the barks (Figure 6), as well as to their most similar group, the fruiting bodies (Figure 7). Only PCA was used to evaluate the distribution of signals in the leaves, cones, and berries since only two species each represented the groups of cones and berries, so statistical evaluation of the separation of these sets was not possible. The metabolomes of the two cones displayed great similarity, shown through the near overlapping of their values on the score plot. The dissimilarity of some of the leaf samples may be attributed to the *G. hispidula* and *V. vitis-idaea* plants having more similar growth forms while the *P. glauca* sample is characterized as a conifer needle.

Major phytochemical signals highly representative of the evaluated groupings in the discriminate analyses separate furthest from the origin, and so are easily determined through visual examination of the plots. These major signals are presented in Table 3, where the first portion of the signal code refers to the retention time of that mass signal, and the second portion refers to the calibrated mass/charge ratio. Signals are also presented with tentative identifications made through analysis using the Metlin MS/MS metabolite database. Whereas phenolic compounds were more frequently identified as markers of leaves and diterpenes were characteristic of barks, markers tentatively identified in fruiting bodies included both phenolics and terpenes.

**TABLE 3:** Key signals (retention time and calibrated mass/charge ratio) identified through evaluation of multivariate analysis loadings figures.

Signal	Association	Tentative ID
Leaves and Barks		
RT7.98_621.3077	Leaf	Protein
RT2.84_303.0504	Leaf	Quercetin derivative
RT3.10_303.0504	Leaf	Quercetin derivative
RT2.90_303.0504	Leaf	Quercetin derivative
RT7.59_593.2763	Leaf	Chlorophyll derivative Pheophorbide A
RT2.09_139.0394	Leaf	Phenolic
RT6.02_299.2009	Bark	Diterpene
Leaves, Cones and Berries		
RT7.98_621.3077	Leaf	Protein
RT7.59_593.2763	Leaf	Chlorophyll derivative Pheophorbide A
RT6.69_313.1073	Leaf	Flavone
RT4.16_107.0498	Leaf	Simple phenol
RT2.09_139.0394	Leaf	Phenolic
RT3.10_303.0504	Leaf	Quercetin derivative
RT0.54_243.0843	Berry	Sulfur compound
RT1.96_287.0554	Berry	Flavonoid
RT6.35_122.0970	Berry	Amino acid derivative
RT6.36_122.0970	Berry	Amino acid derivative
RT6.02_299.2009	Cone	Diterpene
RT6.66_301.2168	Cone	Fatty acid
RT6.70_301.2168	Cone	Fatty acid

Tentative IDs based on evaluation of signal masses in Metlin.scripps online database.

## Discussion

### Discussion of Data

The objective of this study was to explore the relationship between traditional knowledge within a directed set of uses (treatment of diabetes symptoms) and the patterns of effects in relevant bioassays of such medicinal plants. The strongest detected association was between the importance of CEI medicinal plants (through SIV) and their inhibitory activity on CYP enzymes in the herb-drug interaction assays. This is not surprising given that the role of many CYP enzymes is to respond to chemical challenges coming from the environment (Kennedy and Tierney, 2012). Indeed, the expression of several key CYP isozymes involved in drug metabolism (CYP 3A4/5, 1A2, 2D6, 2C9, 2C19 and 1E2) is tightly modulated by xenobiotic sensors of the class of intracellular receptors such as AHR, CAR, PXR, and LXR (Honkakoski and Negishi, 2000). However, our studies did not address interaction of plant extracts with such receptors, nor CYP expression, but rather focused on interference with CYP activity to highlight the potential for herb-drug interactions. Our analysis shows that traditional knowledge appears to favor plants that can reduce CYP-induced xenobiotic metabolism. One interpretation could be that such CYP inhibition may increase the bioavailability of the bioactive components of medicinal plant species, thereby enhancing their potential biological activity.

In addition, a slightly weaker association was also seen between the most important CEI plants in terms of traditional knowledge and radical scavenging activities displayed in the secondary antidiabetic potential assays. Plants rich in antioxidant polyphenols have received much attention for their positive effects on disease prevention (Scalbert et al., 2005), Evaluating a larger collection of CEI medicinal plants, Fraser et al. (2007) identified significant correlations between traditional knowledge and both antioxidant activity and total phenolic content. In contrast, enzyme inhibition across Cree plant extracts did not correlate with phenolic content (Tam et al., 2009).

No strong association patterns were seen among the plant extracts in relation to the primary assays; however, while there does not appear to be a specific universal anti-diabetes plant (not unexpectedly), there was almost always at least one plant that either closely matched or exceeded the activity of pharmacologically relevant positive controls. For instance, *P. glauca* was comparable to insulin in its ability to inhibit the activity of G6Pase in hepatocytes while *L. laricina* was more than twice as effective as insulin at stimulating glycogen synthase activity through GSK-3 phosphorylation (Nachar et al., 2013). Likewise, both *L. laricina* and *R. groenlandicum* were more effective at increasing intracellular triglyceride levels in adipocytes than the PPARλ agonist and anti-diabetes drug rosiglitazone (Spoor et al., 2006).

Similar patterns were seen between the traditional knowledge and the plant pharmacology in terms of the top and bottom plants. The species with the lowest scoring on the active-inactive activity metrics (Figure 4) was *P. balsamifera*. It also was among the lowest five plants for all three PIV categories (Table 2). In Leduc’s original anti-diabetic Cree ethnobotany (Leduc et al., 2006), *P. balsamifera* was not among the species originally identified by the Cree elders in Mistissini as having a significant correlation of use with diabetic symptoms. It was only identified when later interviews were conducted with elders of other nearby Cree communities. On the other hand, the evaluation of syndromic importance identified *R. groenlandicum*, *L. laricina*, and *A. balsamea* as the most important plants to the Cree, based on the symptoms of diabetes. Additionally, these species were identified as some of the most used out of 546 taxa used medicinally in boreal Canada (Uprety et al., 2012). Next to *S. purpurea*, these three species also displayed the highest biological activity in several bioassays. Even though *S. purpurea* ranked much lower in Leduc’s analysis, the consistency of these other three species again highlights the significance of the CEI traditional knowledge.

The similar activities displayed by leaf extracts in the secondary antidiabetic potential assays likely reflect strong phenolic content in leaf parts of bioactive plants. This is consistent with the fact that a higher phenolic content is strongly correlated with both antioxidant (Fraser et al., 2007) and antiglycation (Harris et al., 2011) potential. Comparisons with extracts from common market produce also found that many traditional Boreal forest medicinal plants, including many evaluated here, have significantly higher radical scavenging abilities, and have antioxidant effects comparable to green tea, Vitamin C, and Vitamin E (McCune and Johns, 2002). This is not surprising given the known antioxidant activity of polyphenols (Perron and Brumaghim, 2009; Hussain et al., 2016), many of which are present as major components of leaf material, as seen in our chemical analysis. Accordingly, based on our data, *in vitro* radical scavenging assays such as measures of antioxidant and antiglycation activity appear to better reflect plant part and extract chemistry rather than collected ethnobotanical knowledge. As mentioned above for CYP inhibition, our team failed to uncover a strong relationship between antioxidant capacity and antidiabetic potential over the course of several studies.

A consistent theme in the exploration of these plants’ pharmacological and phytochemical profiles was the dissimilarity of barks. Across all evaluations, the species whose barks were evaluated defied groupings both within themselves and in relation to the other plant part groupings. One way to explain this high variability comes forth when one looks at the chemistry of barks compared to the other plant parts and at the natural diversity of compound classes often associated with different plant parts. Namely, terpenes have been thoroughly analyzed for their role as a major mechanism of defense of conifer tree species (Keeling and Bohlmann, 2006) and, although terpenes are also present in leaves, they are produced in much lesser quantity and diversity (Courtois et al., 2012). The dissimilarity of the barks may also be related more simply to the fact that four plant families were evaluated across the barks, whereas only two were evaluated across the leaves.

Finally, in terms of cone species, *P. banksiana* and *P. mariana* consistently associated with specific groups throughout the analysis. Primarily, their association of activity on stimulating glucose uptake in fat cells was seen. Further, their activity was associated with both adipogenesis and immunomodulatory assays. When looking at the original data (Tam et al., 2009; Liu, 2011; unpublished), the two cones had strong activity associated with inhibition of CYPs 1A2, 2E1, and 4A11, a group of enzymes known for their metabolism of endogenous fatty acids into important signaling molecules (Westphal et al., 2011). Specifically, the CYP generated metabolite of arachidonic acid, epoxyeicosatrienoic acid (EET), has been shown to have cytoprotective effects for maintaining Akt and AMPK signaling in numerous cell lines and preventing insulin resistance *in vivo* in animal models subjected to high fat diets (Xu et al., 2013; He et al., 2016). Moreover, two of the three defining phytochemical signals associated with the cones were tentatively identified as fatty acids. Indeed, it may prove useful to focus bioactive phytochemical compound identification of these two species’ cones to either EET-like fatty acids or other fatty acids that may be metabolized by the aforementioned set of CYP enzymes.

### Challenges and Limitations

Several issues with this data set must be acknowledged. Firstly, our original research used different metrics to identify significant biological activity across bioassays. Some studies determined significance relative to positive controls, whereas others referred to negative controls. Further, some publications quantified these differences with *p*-values while others did not. There were also cases where different post-hoc tests of significance and different sample sizes yielded different distinctions of what plants were considered active or non-active in different publications (Harris, 2008; Harbilas et al., 2009). This is one of the main reasons why it was decided to evaluate the data both as a binary (active vs non-active, according to statistical significance) and as raw data standardized to the most active plant. The first approach allowed the acknowledgment of activity differences through statistical significance, whereas the subsequent approach best represents the degree of differences in activity between each plant.

Secondly, it is important to note that primary antidiabetic potential bioassays were all based on cellular models of insulin-target tissues (liver, muscle, adipose tissue, intestine). In contrast, the majority of secondary antidiabetic bioassays (all but 4) as well as all herb-drug interaction bioassays used acellular models, the latter based on highly defined recombinant enzymes. Despite the fact that standard operating procedures were generally used across all data sets, the use of cell-based models can provide an explanation for the greater dispersion of the primary antidiabetic potential data set and underlie the difficulty in obtaining stronger associations between such data and Cree traditional knowledge in our multivariate statistical analyses. Conversely, data generated using acellular, most often enzyme-based, bioassays yielded much less variable results; likely enhancing the discrimination of plants along a spectrum of biological activities and enabling stronger statistical association with Cree traditional knowledge. Albeit cell-based models are inherently strong because they integrate several biological events that culminate in the measured outcome.

Thirdly, unlike what our team did with the SIV, the PIV used in our analysis considered all bioassays as equivalent in terms of relevant pharmacological activity. Indeed, for the SIV, we had a group of clinical endocrinologists and diabetes researchers provide a rank for the importance or relevance of each symptom in the clinical realm of diabetes; ethnobotanical data being weighed accordingly (Leduc et al., 2006). A similar approach could thus be used to determine which bioassays may reflect better clinical outcomes. This weighing of the clinical relevance of bioassays may have improved the relationships that were hinted between traditional knowledge and bioassays, in particular the ones targeting primary antidiabetic potential.

Finally, our analysis did not take into consideration the period in which plants were collected, nor the geographical ranges of these collections. It must be noted that most of the studies used extracts from uniform collection times and areas. It is well known that rates of phytochemical biosynthesis vary significantly in relation to seasonal and latitudinal variations in light intensity, climate, and soil composition (Jaakola and Hohtola, 2010). Our team confirmed similar and relevant comments from Cree Elders about the variability of “strength” of given plants according to their origin within Eeyou Istchee. Indeed, previous studies demonstrated significant latitudinal changes in the phenolic content of Labrador tea that could be related to photoperiod (Rapinski et al., 2014) and in turn affected the plant’s biological activity (Rapinski et al., 2015). As a result, it must be acknowledged that the results discussed here do not fully represent the diversity of activity that may be experienced by members of different communities throughout the Eeyou Istchee territory. Instead, the methods explored here serve to shed light on some of the promises and pitfalls to be expected from this approach in future research.

## Conclusion

One of the purposes of this investigation was to explore the power and potential for multivariate analysis of multidisciplinary data in the context of ethnopharmacology. A useful application of the work would have been to develop predictive models where the general activities of a given plant could be projected based on its taxonomic family, the part used, or a quick screen of its phytochemical profile. Perhaps unsurprisingly, despite the patterns discussed here, it is apparent that there was still quite a long way to go before a model of such power can be fully realized. These results, however, can inform the design of future studies with fewer limitations and greater statistical power for investigating potential relationships between ethnobotany, phytochemistry, and pharmacology.

Overall, this work highlights the depth and insight of traditional knowledge within indigenous communities with regards to plant medicines. Specifically, CYP inhibition patterns as a whole best reflected patterns of traditional medicine importance in the CEI traditional knowledge, whereas potent primary bioactivities were seen in individual plants determined to be most important to the CEI for anti-diabetes purposes. In the secondary anti-diabetes assays, pharmacological variability was better described by plant biology, mostly in terms of the plant part used. Thus, traditional indigenous knowledge should be cherished for its ability to guide the deciphering of plant qualities that may have previously been overlooked by science.

## Data Availability

The datasets generated for this study are available on request to the corresponding author.

## References

[B1] Chambers M. C. MacLean B. Burke R. Amodei D. Ruderman D. L. Neumann S. (2012). A Cross-Platform Toolkit for Mass Spectrometry and Proteomics. Nat. Biotechnol. 30, 918–920. 10.1038/nbt.2377 23051804 PMC3471674

[B2] Chandler R. F. Hooper S. N. Harvey M. J. (1982). Ethnobotany and Phytochemistry of yarrow,Achillea millefolium, Compositae. Econ. Bot. 36, 203–223. 10.1007/BF02858720

[B3] Core E. L. (1967). Ethnobotany of the Southern Appalachian Aborigines. Econ. Bot. 21, 199–214. 10.1007/BF02860370

[B4] Courtois E. A. Baraloto C. Paine C. E. Petronelli P. Blandinieres P. A. Stien D. (2012). Differences in Volatile Terpene Composition between the Bark and Leaves of Tropical Tree Species. Phytochemistry 82, 81–88. 10.1016/j.phytochem.2012.07.003 22863563

[B5] Fabricant D. S. Farnsworth N. R. (2001). The Value of Plants Used in Traditional Medicine for Drug Discovery. Environ. Health Perspect. 109, 69–75. 10.1289/ehp.01109s169 11250806 PMC1240543

[B6] Fraser M. H. Cuerrier A. Haddad P. S. Arnason J. T. Owen P. L. Johns T. (2007). Medicinal Plants of Cree Communities (Québec, Canada): Antioxidant Activity of Plants Used to Treat Type 2 Diabetes Symptoms. Can. J. Physiol. Pharmacol. 85, 1200–1214. 10.1139/Y07-108 18066122

[B7] Haddad P. S. Musallam L. Martineau L. C. Harris C. Lavoie L. Arnason J. T. (2012). Comprehensive Evidence-Based Assessment and Prioritization of Potential Antidiabetic Medicinal Plants: A Case Study from Canadian Eastern James Bay Cree Traditional Medicine. Evidence-Based Complement. Altern. Med. 2012, 1–14. 10.1155/2012/893426 PMC3247006 22235232

[B8] Harbilas D. Martineau L. C. Harris C. S. Adeyiwola-Spoor D. C. Saleem A. Lambert J. (2009). Evaluation of the Antidiabetic Potential of Selected Medicinal Plant Extracts from the Canadian Boreal forest Used to Treat Symptoms of Diabetes: Part II. Can. J. Physiol. Pharmacol. 87, 479–492. 10.1139/Y09-029 19526043

[B9] Harris C. S. Beaulieu L. P. Fraser M. H. McIntyre K. L. Owen P. L. Martineau L. C. (2011). Inhibition of Advanced Glycation End Product Formation by Medicinal Plant Extracts Correlates with Phenolic Metabolites and Antioxidant Activity. Planta Med. 77, 196–204. 10.1055/s-0030-1250161 20717877

[B10] Harris C. S. (2008). “Anti-diabetic and Neuroprotective Activities of Phytochemicals in Traditionally Used Boreal Plants,”ProQuest Diss. Theses. Ottawa, ON: uO Reserch. Available at: : http://search.proquest.com.ezp-prod1.hul.harvard.edu/docview/911791953?accountid=11311%5Cnhttp://sfx.hul.harvard.edu/sfx_local?url_ver=Z39.88-2004&rft_val_fmt=info:ofi/fmt:kev:mtx:dissertation&genre=dissertations+&+theses&sid=ProQ.

[B11] He J. Wang C. Zhu Y. Ai D. (2016). Soluble Epoxide Hydrolase: A Potential Target for Metabolic Diseases. J. Diabetes 8, 305–313. 10.1111/1753-0407.12358 26621325

[B12] Höft M. Barik S. K. Lykke A. M. (1999). Quantitative Ethnobotany Applications of Multivariate and Statistical Analyses in Ethnobotany. Paris, France: UNESCO. Available at: http://pure.au.dk/portal/files/17477574/hoeftbariklykke1999.pdf (Accessed November 16, 2018).

[B13] Honkakoski P. Negishi M. (2000). Regulation of Cytochrome P450 (CYP) Genes by Nuclear Receptors. Biochem. J. 347, 321–337. 10.1042/bj3470321 10749660 PMC1220963

[B14] Hussain T. Tan B. Yin Y. Blachier F. Tossou M. C. B. Rahu N. (2016). Oxidative Stress and Inflammation: What Polyphenols Can Do for Us? Oxidative Med. Cell Longevity 2016, 1–9. 10.1155/2016/7432797 PMC5055983 27738491

[B15] Jaakola L. Hohtola A. (2010). Effect of Latitude on Flavonoid Biosynthesis in Plants. Plant Cel Environ 33, 1239–1247. 10.1111/j.1365-3040.2010.02154.x 20374534

[B16] Keeling C. I. Bohlmann J. (2006). Genes, Enzymes and Chemicals of Terpenoid Diversity in the Constitutive and Induced Defence of Conifers against Insects and Pathogens. New Phytol. 170, 657–675. Available at: https://journals-scholarsportal-info.proxy.bib.uottawa.ca/pdf/0028646x/v170i0004/657_geacotocaiap.xml. (Accessed January 24, 2019). 10.1111/j.1469-8137.2006.01716.x 16684230

[B17] Kennedy C. J. Tierney K. B. (2012). “Xenobiotic Protection Xenobiotic protection/Resistance Mechanisms in Organisms,” in Encyclopedia of Sustainability Science and Technology (New York, NY: Springer New York), 12293–12314. 10.1007/978-1-4419-0851-3_51

[B18] Leduc C. Coonishish J. Haddad P. Cuerrier A. (2006). Plants Used by the Cree Nation of Eeyou Istchee (Quebec, Canada) for the Treatment of Diabetes: A Novel Approach in Quantitative Ethnobotany. J. Ethnopharmacol. 105, 55–63. 10.1016/j.jep.2005.09.038 16297584

[B36] Liu R. (2011). Effects of Selected Natural Health Products on Drug Metabolism: Implications for Pharmacovigilance. Master thesis. Canada: University of Ottawa.

[B19] McCune L. M. Johns T. (2002). Antioxidant Activity in Medicinal Plants Associated with the Symptoms of Diabetes Mellitus Used by the Indigenous Peoples of the North American Boreal forest. J. Ethnopharmacol. 82, 197–205. 10.1016/S0378-8741(02)00180-0 12241996

[B20] Nachar A. Vallerand D. Musallam L. Lavoie L. Badawi A. Arnason J. (2013). The Action of Antidiabetic Plants of the Canadian James Bay Cree Traditional Pharmacopeia on Key Enzymes of Hepatic Glucose Homeostasis. Evid. Based Complement. Alternat Med. 2013, 189819–9. 10.1155/2013/189819 23864882 PMC3707264

[B21] Perron N. R. Brumaghim J. L. (2009). A Review of the Antioxidant Mechanisms of Polyphenol Compounds Related to Iron Binding. Cell Biochem. Biophys. 53, 75–100. 10.1007/s12013-009-9043-x 19184542

[B22] Pluskal T. Castillo S. Villar-Briones A. Oresic M. (2010). MZmine 2: Modular Framework for Processing, Visualizing, and Analyzing Mass Spectrometry-Based Molecular Profile Data. BMC Bioinformatics 11, 395. 10.1186/1471-2105-11-395 20650010 PMC2918584

[B23] Prance G. T. (1991). What Is Ethnobotany Today? J. Ethnopharmacol. 32, 209–216. 10.1016/0378-8741(91)90120-3 1881159

[B24] Rapinski M. Liu R. Saleem A. Arnason J. T. Cuerrier A. (2014). Environmental Trends in the Variation of Biologically Active Phenolic Compounds in Labrador tea, *Rhododendron Groenlandicum,* from Northern Quebec, Canada. Botany 92, 783–794. 10.1139/cjb-2013-0308

[B25] Rapinski M. Musallam L. Arnason J. T. Haddad P. Cuerrier A. (2015). Adipogenic Activity of Wild Populations ofRhododendron Groenlandicum, a Medicinal Shrub from the James Bay Cree Traditional Pharmacopeia. Evidence-Based Complement. Altern. Med. 2015, 1–7. 10.1155/2015/492458 PMC4609817 26508979

[B26] Rymer L. (1976). The History and Ethnobotany of Bracken. Bot. J. Linn. Soc. 73, 151–176. 10.1111/j.1095-8339.1976.tb02020.x

[B27] Scalbert A. Manach C. Morand C. Rémésy C. Jiménez L. (2005). Dietary Polyphenols and the Prevention of Diseases. Crit. Rev. Food Sci. Nutr. 45, 287–306. 10.1080/1040869059096 16047496

[B28] Schultes R. E. (1994). Amazonian Ethnobotany and the Search for New Drugs. Available at: https://onlinelibrary-wiley-com.proxy.bib.uottawa.ca/doi/pdf/10.1002/9780470514634.ch8 (Accessed November 16, 2018). 10.1002/9780470514634.ch8 7736849

[B29] Shang N. Saleem A. Musallam L. Walshe-Roussel B. Badawi A. Cuerrier A. (2015). Novel Approach to Identify Potential Bioactive Plant Metabolites: Pharmacological and Metabolomics Analyses of Ethanol and Hot Water Extracts of Several Canadian Medicinal Plants of the Cree of Eeyou Istchee. PLoS One 10, e0135721. 10.1371/journal.pone.0135721 26263160 PMC4532419

[B30] Spoor D. C. Martineau L. C. Leduc C. Benhaddou-Andaloussi A. Meddah B. Harris C. (2006). Selected Plant Species from the Cree Pharmacopoeia of Northern Quebec Possess Anti-diabetic Potential. Can. J. Physiol. Pharmacol. 84, 847–858. 10.1139/y06-018 17111029

[B31] Tam T. W. Liu R. Arnason J. T. Krantis A. Staines W. A. Haddad P. S. (2009). Actions of Ethnobotanically Selected Cree Anti-diabetic Plants on Human Cytochrome P450 Isoforms and Flavin-Containing Monooxygenase 3. J. Ethnopharmacol. 126, 119–126. 10.1016/j.jep.2009.07.036 19665535

[B32] Turner N. C. Bell M. A. M. (1971). The Ethnobotany of the Coast Salish Indians of Vancouver Island. Econ. Bot. 25, 63–99. 10.1007/BF02894564

[B33] Uprety Y. Asselin H. Dhakal A. Julien N. (2012). Traditional Use of Medicinal Plants in the Boreal forest of Canada: Review and Perspectives. J. Ethnobiol. Ethnomed. 8, 7. 10.1186/1746-4269-8-7 22289509 PMC3316145

[B34] Westphal C. Konkel A. Schunck W. H. (2011). CYP-eicosanoids--a New Link between omega-3 Fatty Acids and Cardiac Disease? Prostaglandins Other Lipid Mediat 96, 99–108. 10.1016/j.prostaglandins.2011.09.001 21945326

[B35] Xu X. Tu L. Feng W. Ma B. Li R. Zheng C. (2013). CYP2J3 Gene Delivery Up-Regulated Adiponectin Expression via Reduced Endoplasmic Reticulum Stress in Adipocytes. Endocrinology 154, 1743–1753. 10.1210/en.2012-2012 23515292

