# Supplementary material for: A Multivariate Approach to Ethnopharmacology: Antidiabetic Plants of Eeyou Istchee
Source: Front Pharmacol. 2022 Jan 18;12:511078. doi: 10.3389/fphar.2021.511078 (PMC8808167; doi:10.3389/fphar.2021.511078)
Supplement: Supplementary file 1 [file DataSheet1.DOCX]

Supplementary Material

**Supplementary Table 1.** List and description of bioasays included in the pharmacology meta-analysis.

| Bioassay (Code) | Endpoint | Positive Control | Cell Type | Reference |
| --- | --- | --- | --- | --- |
| *Primary antidiabetic bioassays* | | | | |
| Inhibition of hepatocyte G6Pase (G6Pase) | Glucose release into medium after incubation | Insulin | H4IIE | Nachar et al., 2013 |
| Activation of hepatocyte Akt (Hep Akt) | Ratio of phosphorylated Akt to total Akt after incubation | Insulin | H4IIE | Nachar et al., 2013 |
| Activation of hepatocyte AMPK (Hep AMPK) | Ratio of phosphorylated AMPK to total AMPK after incubation | AICAR | H4IIE | Nachar et al., 2013 |
| Activation of hepatocyte Glycogen Synthase (GS) | Ratio of phosphorylated GS to total GS after incubation | Insulin/AICAR | H4IIE | Nachar et al., 2013 |
| Deactivation of hepatocyte GSK-3 (GSK-3) | Ratio of phosphorylated GSK-3 to total GSK-3 after incubation | Insulin/AICAR | H4IIE | Nachar et al., 2013 |
| Stimulation of glucose uptake into muscle cells (MSA, MSN, MSS, MLA, MLN, MLS) | Radioactive glucose uptake by muscle (M) cells after short- (S) or long-term (L) incubation with plant extracts in the absence (A) or presence of physiological (N, normal) or supraphysiological (S) concentrations of insulin | Metformin | C2C12 | Spoor et al., 2006; Harbilas et al., 2009 |
| Stimulation of glucose uptake into adipose cells (ASA, ASN, ASS, ALA, ALN, ALS) | Radioactive glucose uptake by adipose (A) cells after short- (S) or long-term (L) incubation with plant extracts in the absence (A) or presence of physiological (N, normal) or supraphysiological (S) concentrations of insulin | Fenugreek | 3T3-L1 | Spoor et al., 2006; Harbilas et al., 2009 |
| Effect on adipogenesis (Adipogenesis) | Ratio of intracellular triglyceride content before and after incubation | Rosiglitazone | 3T3-L1 | Spoor et al., 2006; Harbilas et al., 2009 |
| Instantaneous inhibition of glucose uptake into intestinal cells (ISA) | Inhibition of radioactive glucose uptake by intestinal (I) cells after short-term (S – 10 min) incubation with plant extracts in the absence (A) of insulin | Phloretin/Phlorizin | Caco-2 Monolayer | Nistor Baldea et al., 2010 |
| Chronic inhibition of glucose uptake into intestinal cells (ILA) | Inhibition of radioactive glucose uptake by intestinal (I) cells after long-term (L – 6 hr) incubation with plant extracts in the absence (A) of insulin | Phloretin/Phlorizin | Caco-2 Monolayer | Nistor Baldea et al., 2010 |
| *Secondary antidiabetic bioassays* | | | | |
| Antioxidant Capacity (DPPH) | Colorimetric evaluation to determine degree of quenching of the DPPH free radical | Catechin/Epicatechin/  Quercetin | Cell-free | Fraser et al., 2007 |
| Inhibition of lipid peroxidation (Conjugated diene - CD)  Inhibition of lipid peroxidation (TBARS) | Prolongation of lag time before change in absorbance; indicates diene conjugation with LDL | Ascorbic acid/Trolox | Cell-free | Fraser et al., 2007 |
|  | Fluorescence of TBARS over 4 hour incubation of LDL with plant extracts | Ascorbic acid/Trolox | Cell-free | Fraser et al., 2007 |
| Oxygen radical absorbance capacity (ORAC) | Persistence of fluorescent signal through quenching of the ROS generating compound AAPH. | Quercetin/Trolox | Cell-free | Harris et al., 2011 |
| Cellular Protection (GluTox, GluDep) | Cell proliferation, as measured by fluorescence after WST-1 addition, after 96h in media with high glucose concentration | Vehicle + high/low glucose | PC12-AC | Harbilas et al., 2009 |
|  | Cell proliferation, as measured by fluorescence after WST-1 addition, after 96h in media with low glucose concentration | Vehicle + high/low glucose | PC12-AC | Harbilas et al., 2009 |
| Advanced Glycation Endproduct Inhibition (AGEs) | Fluorescence of serum/sugar/extract mix after incubation | Quercetin | Cell-free | Harris et al., 2011 |
| CML-BSA Adduct Formation Inhibition (CML-BSA) | Immunostaining and quantification of separated protein content | Quercetin | Cell-free | Harris et al., 2011 |
| Aldose Reductase Inhibition (Aldose) | Fluorescence of NADPH oxidation in presence of aldose reductase/extract/glucose mix | Quercetin | Cell-free | Nguyen, 2011 |
| Anti-inflammatory Activity | TNF-α content after incubation with extract and LPS stimulation | Parthenolide | THP-1 monocytes | Walshe-Roussel, 2014 |
| Pro-inflammatory Activity | TNF-α content after incubation with extract | Echinacea extract | THP-1 monocytes | Walshe-Roussel, 2014 |
| *Safety* | | | | |
| Human Carboxylesterase Inhibition (Human Carboxylesterase) | Spectrographic quantification of oseltamivir metabolites  After incubation with extracts | Vehicle | Human Liver Microsomes | Liu, 2011 |
| Flavin-containing Monooxygenase-3 Inhibition (FMO3) | Spectrographic quantification of MpTS metabolites after incubation with extracts | Vehicle | Cell-free | Liu, 2011 |
| Cytochrome P450 Inhibition (CYP1A2, CYP2B6, CYP2C8, CYP2C9, CYP2C19, CYP2D6, CYP2E1, CYP19, CYP3A4, CYP3A7, CYP4A11) | Fluorescence of metabolized enzyme substrates after incubation with extracts | Furafylline (1A2), Tranylcypromine (2B6, 2C19), Ketoconazole (2C8, 3A4, 3A7), Sulfaphenazole (2C9), Quinidine (2D6), Diethyldithiocarbamate (2E1), Bifonazole (19) | Cell-free | Tam et al., 2009 |

**Supplementary Table 2.** Summary of studies that generated the data used in this analysis. The same original plant material was used across all studies and methods for collection and preparation to freeze dried extracts is outlined in Spoor et al. (2006) and Harbilas et al. (2009) for the source materials. Individual studies targeted different bioactivities across multiple cell lines (**Sup Table 1**) where maximal non-toxic concentration of plant extract was used in each case.

| Study | Plant Investigated | Chemical Analysis |
| --- | --- | --- |
| Beaulieu et al. (2010) | *Vaccinium vitis-idaea* L. | Y – HPLC |
| Cieniak et al. (2015) | *Sarracenia purpurea* L. | Y – UPLC |
| Eid et al. (2016) | *Rhododendron groenlandicum* (Oeder) Kron & Judd | Y – HPLC |
| Fraser et al. (2007) | All* | N |
| Guerrero-Analco et al. (2010) | *Sorbus decora* (Sarg.) C. K. Schneid | Y – HPLC |
| Guerrero-Analco et al. (2014) | *Sorbus decora* (Sarg.) C. K. Schneid*, Larix laricina* Du Roi (K. Koch)*, Sarracenia purpurea* L.*, Alnus incana* (Du Roi) R. T.*, Populus balsamifera* L.*, Vaccinium vitis-idaea* L.*, Rhododendron groenlandicum* (Oeder) Kron & Judd*, Rhododendron tomentosum* (Harjama) | Y – HPLC |
| Harbilas et al. (2009) | *Gaultheria hispidula* (L.) Muhl.*, Juniperus communis* L.*, Kalmia angustifolia* L.*, Lycopodium clavatum* L.*, Picea glauca* (Moench.) Voss*, Populus balsamifera* L.*, Rhododendron tomentosum* (Harjama)*, Salix planifolia* Pursh*, Vaccinium vitis-idaea* L. | Y – HPLC |
| Harris et al. (2008) | *Picea glauca* (Moench.) Voss | Y – HPLC |
| Harris et al. (2011) | All | N |
| Martineau et al. (2010) | *Alnus incana subsp. rugose* (Du Roi) R. T.*, Populus balsamifera* L. | Y – HPLC |
| Nachar et al. (2013) | All | N |
| Nistor Baldaea et al. (2010) | All | N |
| Saleem et al. (2010) | *Rhododendron groenlandicum* (Oeder) Kron & Judd*, Rhododendron tomentosum* (Harjama) | Y – HPLC |
| Shang et al. (2012) | *Larix laricina* Du Roi (K. Koch) | Y – HPLC |
| Spoor et al. (2006) | *Abies balsamea* (L.) Mill.*, Alnus incana subsp. rugose* (Du Roi) R. T.*, Larix laricina* Du Roi (K. Koch)*, Picea mariana* (P. Mill) BSP*, Pinus banksiana* Lamb.*, Rhododendron groenlandicum* (Oeder) Kron & Judd*, Sarracenia purpurea* L.*, Sorbus decora* (Sarg.) C. K. Schneid | Y – HPLC |
| Tam et al. (2009) | All | N |

*All 17 CEI plants covered in this study, outlined in **Table 1**.

**Supplementary Table 3.** Phytochemical characterizations of traditional CEI medicinal plant hydroethanolic extracts taken from studies under the TAAM.

| Species | Study | Marker Compounds |
| --- | --- | --- |
| *A. balsamea* | Spoor et al. (2006) | p-Coumaric acid  Gallocatechin |
| *A. incana* | Martineau et al. (2010) | Rubranoside A  1,7-bis-(3,4-dihydroxyphenyl)-5-hydroxyheptane-3-O-β-D-xylopyranoside  Oregonin  Alnuside A  Alnuside B  Oregonin  Catechin  Hirustanone  Hirustanonol |
| *G. hispidula* | Harbilas et al. (2009) | Chlorogenic acid  Catechins  Taxifolin  Myricetin  Quercetin glycosides |
| *J. communis* | Harbilas et al. (2009) | Catechins  Kaempferol  Quercetin glycosides |
| *K. angustifolia* | Harbilas et al. (2009) | Catechins  A-type procyanidin  Quercetin glycosides |
| *L. laricina* | Shang et al. (2012) | 23-oxo-3-hydroxycycloart-24-en-26-oic acid  13-Epitorulosol  13-Epicupressic acid  19-norlabda-8(17),14-dien-4,13-diol  Lariciresinol-3-acetate  Lariciresinol-coumarate  Rhapontigenin  Piceatannol  Rhaponticin  Taxifolin |
| *L. clavatum* | Harbilas et al. (2009) | Ferulic acid derivatives  Apigenin derivatives |
| *P. glauca* | Harris et al. (2008) | Benzoic acid derivative  Tetrahydroxystilbene  Methoxytrihydroxystilbene  Catechin  Taxifolin  Quercetin glycoside  Kaempferol glycoside  Isorhamnetin glycoside |
| *P. mariana* | Spoor et al. (2006) | p-Coumaric acid  Hydroxystilbenes |
| *P. banksiana* | Spoor et al. (2006) | Taxifolin  Catechin  Procyanidins |
| *P. balsamifera* | Martineau et al. (2010) | Salicin  Salicortin  Coumaroylglucoside  Populoside  Salireposide  Trichocarposide |
| *R. groenlandicum* | Saleem et al. (2010) | (+)-Catechin  (−)-Epicatechin  Chlorogenic acid  Myricitin  Procyanidin B2  Procyanidin A1  Quercetin-3-O-glucoside  Quercetin-3-O-galactoside  Rutin  Quercetin-3-O-rhamnoside |
| *R. tomentosum* | Saleem et al. (2010) | Chlorogenic acid  p-Coumaric acid  Myricetin  Quercetin  Quercetin 3-O-galactoside  Quercetin 3-O-glucoside  Quercetin pentoside  Quercetin 3-O-rhamnoside  Caffeic acid derivatives  (+)-Catechin  Procyanidin B1  Procyanidin B2  Procyanidin B3 |
| *S. planifolia* | Harbilas et al. (2009) | Salicin  Isosalireposide derivatives  Tremulacin |
| *S. purpurea* | Cieniak et al. (2015) | (+)-Catechin  Morroniside  (-)-Epicatechin  Taxifolin-3-O-glucoside  Betulinic acid  Ursolic acid  Quercetin-3-O-galactoside  Rutin  Quercetin-3-O-glucoside Kaempferol-3-O-rutinoside |
| *S. decora* | Guerrero-Analco et al. (2010) | 23,28-Dihydroxyursen-12-ene-3β-caffeate  23,28-Dihydroxylupan-12-ene-3β-caffeate  3β,23,28-Trihydroxy-12-ursene  23-Hydroxybetulin  Uvaol  Betulin  R-Amyrin  Betulinic acid  (+)-Catechin,  (-)-Epicatechin |
| *V. vitis-idaea* | Beaulieu et al. (2010) | Catechin  p-Coumaric acid derivative  Cyanidin-3-O-galactoside  Cyanidin-3-O-glucoside  Cyanidin-3-O-arabinoside  p-Coumaric acid  Quercetin-3-O-galactoside  Quercetin-3-O-glucoside |
